# A combined predictive model based on radiomics features and clinical factors for disease progression in early-stage non-small cell lung cancer treated with stereotactic ablative radiotherapy

**DOI:** 10.3389/fonc.2022.967360

**Published:** 2022-08-02

**Authors:** Hong Yang, Lin Wang, Guoliang Shao, Baiqiang Dong, Fang Wang, Yuguo Wei, Pu Li, Haiyan Chen, Wujie Chen, Yao Zheng, Yiwei He, Yankun Zhao, Xianghui Du, Xiaojiang Sun, Zhun Wang, Yuezhen Wang, Xia Zhou, Xiaojing Lai, Wei Feng, Liming Shen, Guoqing Qiu, Yongling Ji, Jianxiang Chen, Youhua Jiang, Jinshi Liu, Jian Zeng, Changchun Wang, Qiang Zhao, Xun Yang, Xiao Hu, Honglian Ma, Qixun Chen, Ming Chen, Haitao Jiang, Yujin Xu

**Affiliations:** ^1^ Department of Radiology, Cancer Hospital of the University of Chinese Academy of Sciences (Zhejiang Cancer Hospital), Institute of Basic Medicine and Cancer (IBMC), Chinese Academy of Sciences, Hangzhou, China; ^2^ Shaoxing University School of Medicine, Shaoxing, China; ^3^ Department of Radiation Oncology, Sun Yat-sen University Cancer Center, State Key Laboratory of Oncology in South China, Collaborative Innovation Center for Cancer Medicine, Sun Yat-sen University, Guangzhou, China; ^4^ Precision Health Institution, General Electric (GE) Healthcare, Hangzhou, China; ^5^ Department of Radiation Physics, Cancer Hospital of the University of Chinese Academy of Sciences (Zhejiang Cancer Hospital), Institute of Basic Medicine and Cancer (IBMC), Chinese Academy of Sciences, Hangzhou, China; ^6^ Department of Radiation Oncology, Cancer Hospital of the University of Chinese Academy of Sciences (Zhejiang Cancer Hospital), Institute of Basic Medicine and Cancer (IBMC), Chinese Academy of Sciences, Hangzhou, China; ^7^ Department of Thoracic Surgery, Cancer Hospital of the University of Chinese Academy of Sciences (Zhejiang Cancer Hospital), Institute of Basic Medicine and Cancer (IBMC), Chinese Academy of Sciences, Hangzhou, China

**Keywords:** non-small cell lung cancer, stereotactic ablative radiotherapy, progression, radiomics, predictive model

## Abstract

**Purpose:**

To accurately assess disease progression after Stereotactic Ablative Radiotherapy (SABR) of early-stage Non-Small Cell Lung Cancer (NSCLC), a combined predictive model based on pre-treatment CT radiomics features and clinical factors was established.

**Methods:**

This study retrospectively analyzed the data of 96 patients with early-stage NSCLC treated with SABR. Clinical factors included general information (e.g. gender, age, KPS, Charlson score, lung function, smoking status), pre-treatment lesion status (e.g. diameter, location, pathological type, T stage), radiation parameters (biological effective dose, BED), the type of peritumoral radiation-induced lung injury (RILI). Independent risk factors were screened by logistic regression analysis. Radiomics features were extracted from pre-treatment CT. The minimum Redundancy Maximum Relevance (mRMR) and the Least Absolute Shrinkage and Selection Operator (LASSO) were adopted for the dimensionality reduction and feature selection. According to the weight coefficient of the features, the Radscore was calculated, and the radiomics model was constructed. Multiple logistic regression analysis was applied to establish the combined model based on radiomics features and clinical factors. Receiver Operating Characteristic (ROC) curve, DeLong test, Hosmer-Lemeshow test, and Decision Curve Analysis (DCA) were used to evaluate the model’s diagnostic efficiency and clinical practicability.

**Results:**

With the median follow-up of 59.1 months, 29 patients developed progression and 67 remained good controlled within two years. Among the clinical factors, the type of peritumoral RILI was the only independent risk factor for progression (*P*< 0.05). Eleven features were selected from 1781 features to construct a radiomics model. For predicting disease progression after SABR, the Area Under the Curve (AUC) of training and validation cohorts in the radiomics model was 0.88 (95%CI 0.80-0.96) and 0.80 (95%CI 0.62-0.98), and AUC of training and validation cohorts in the combined model were 0.88 (95%CI 0.81-0.96) and 0.81 (95%CI 0.62-0.99). Both the radiomics and the combined models have good prediction efficiency in the training and validation cohorts. Still, DeLong test shows that there is no difference between them.

**Conclusions:**

Compared with the clinical model, the radiomics model and the combined model can better predict the disease progression of early-stage NSCLC after SABR, which might contribute to individualized follow-up plans and treatment strategies.

## Introduction

Lung cancer is the second incidence of diagnosed tumor and is the primary leading cause of cancer-related deaths worldwide (1). Non-small cell lung cancer (NSCLC) accounts for 80-85% of lung cancer. Currently, surgery remains the standard of care for NSCLC (2). For patients who are medically inoperable due to their existing severe chronic disease or their rejection of surgery, the treatment of stereotactic ablative radiotherapy (SABR) has been established as the standard alternative therapy (3–5).

SABR is a non-invasive external beam radiation modality which could facilitate the delivery of ablative doses to the tumor, sparing the surrounding normal tissues over a limited number of fractions. Previous studies had shown that the local control rate could reach 85%~98%, and the 3-year overall survival (OS) can get 48%~65% after SABR in early-stage NSCLC (6–8). However, the patients still had the risk of locoregional recurrence (4%~14%) and distant metastasis (13%~23%) after SABR (9, 10), which is a great challenge for clinicians. Chemotherapy is not ideal because most patients who receive SABR cannot take the risk of surgery due to poor cardiopulmonary function and aged physical condition. In the era of immunotherapy, Immune Checkpoint Inhibitors (ICI) have represented a revolution in treating various stages of NSCLC. The addition of ICI to SABR seems promising, and several multicenter, prospective, randomized controlled clinical trials are underway. A systematic literature review indicated that the ICI-SABR combination has a good safety profile and achieves high rates of local control and greater chances of obtaining abscopal responses than SABR alone, with a relevant impact on progression-free survival (PFS) (11). However, most patients with early-stage NSCLC could be cured after SABR alone, and they shall be waived from the suffering of the harm of systematic therapy. For this reason, finding out those patients will have a high risk of disease progression is becoming essential. Therefore, establishing an effective predictive model to assess the risk of progression and survival probability of early-stage NSCLC patients is of great significance for treatment plan selection or the individual design of follow-up.

Resulting from the heterogeneity of tumors, the growth rate, invasive ability, drug sensitivity, and prognosis of tumors can be different, and the divergence can limit the usefulness of molecular testing-based tissue biopsies (12). Radiomics extracts quantitative features from Computed Tomography (CT), Magnetic Resonance Imaging (MRI), Positron Emission Tomography (PET), and other medical images with high throughput by utilizing computer software (13). Through statistical or computer learning methods, the characteristics most related to clinical results are selected to establish models, which can provide valuable predictive information for the diagnosis and treatment of diseases, and can provide information on tumor cells more comprehensively, systematically, and deeply (12–17). In this study, the radiomics method was used to deeply mine the pre-treatment CT radiomics features, combined with clinical factors, to construct and validate a predictive model for the disease progression of early-stage NSCLC after SABR, providing a feasible and practical reference for clinical guidance of individualized treatment of patients.

## Materials and methods

This retrospective study was approved by the ethics committees of Cancer Hospital of the University of Chinese Academy of Sciences (Zhejiang Cancer Hospital). The requirement for informed consent was waived.

### Patient data

The clinical and imaging data of patients with early-stage NSCLC treated with SABR in the Department of Thoracic Radiation Oncology, Cancer Hospital of the University of Chinese Academy of Sciences from 2012 to 2018 were collected. General information (e.g. gender, age, KPS, Charlson score, lung function, smoking status), pre-treatment lesion status (e.g. diameter, location, pathological type, T stage), radiation parameters (biological effective dose, BED), and the type of peritumoral radiation-induced lung injury (RILI) was classified.

Inclusion criteria: 1) Pathologically confirmed primary NSCLC by bronchoscopy or percutaneous CT-guided biopsy; 2) The TNM clinical stage I~II according to the American Joint Committee on Cancer (AJCC) (8th edition); 3) Have not received other prior antitumor therapy; 4) Thorax CT examination performed before treatment and every 3-6 months follow-up after SABR. Exclusion criteria: 1) coexisting with other primary malignant tumors; 2) incomplete clinical and imaging data; 3) lesions cannot be accurately segmented (e.g. the lesion and peripheral atelectasis cannot be accurately segmented.); 4) lost to follow-up.

### SABR treatment

All patients performed four-dimensional CT simulations with free breathing. The Internal Gross Target Volume (IGTV) was derived from the Maximum Intensity Projection (MIP) of 4DCT and the Planning Target Volume (PTV) was expanded by a 5-mm margin in all directions around the IGTV. The total radiation dose and fraction dose were determined by the radiation oncologists based on the lesion location, volume, and peripheral organs at risk. Target delineation, conformity, and dose limitations in normal tissues were referred to the American Radiation Therapy Oncology Group (RTOG) 0236 study (18). The prescription dose was 5-15Gy per fraction, once a day, with a total dose of 40-70Gy. The BED was calculated using the formula, BED_α/β_ = nd (1+ d/α/β), where n=number of fractions, d=dose per fraction, and α/β=10 Gy for the lung cancer.

### Follow-up

All patients underwent enhanced thorax CT examination one month after the end of treatment and every three months thereafter, and every six months after two years. If progression is suspected, PET-CT or pathological biopsy is performed. Enhanced thorax CT was performed with GE 64-slice CT or Siemens 64-slice CT, tube current 100~300mAs, tube voltage 120 kV, pitch 5.0 mm, slice thickness 5.0 mm. The contrast agent was selected from Opitiray (Ioversol) or Ultravist (Iopromide), and the high-pressure syringe was injected rapidly through the dorsal vein of the hand, the injection rate was 2.5ml/s, and the dose was 80-95ml. Enhanced thorax CT examination was performed 38s after contrast agent injection. Disease progression within two years of follow-up was defined as a high-risk group, and progression or no progression for more than two years was defined as a low-risk group.

### Radiomic analysis

**Figure 1:**
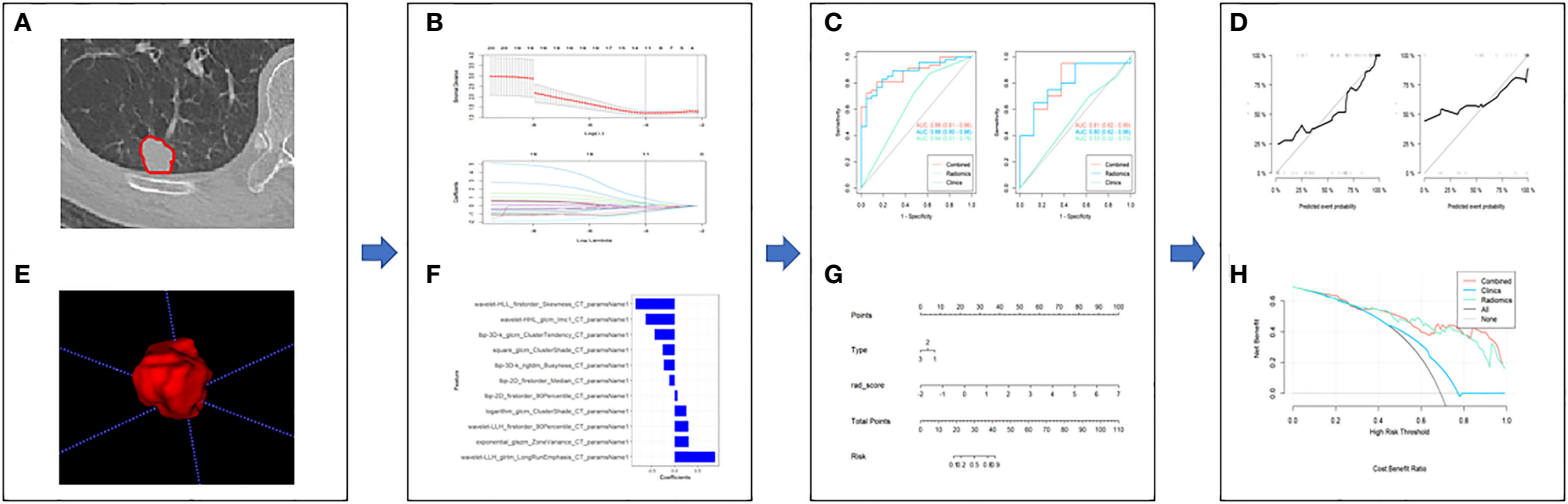
The framework for the radiomics workflow. **(A, B)** Medical imaging segmentation; **(C, D)** Feature extraction and selection; **(E, F)** The ROC curves and nomogram; **(G, H)** Hosmer-Lemeshow Test and the decision curve.

The workflow of the study was shown in **Figure 1**.

### Medical imaging segmentation

The lung-window CT images (window width of 1600 Hounsfield units (HU) and window level of -450 HU; DICM format) of early-stage NSCLC patients before SABR treatment were imported into ITK-SNAP software (Version 3.4.0, http://www.itksnap.org/). A region of interest (ROI) was manually delineated layer by layer by an attending radiologist (who had 10 years of experience with thorax CT images), and a Volume of Interest (VOI) was synthesized. Adjacent aorta, ribs, and pulmonary bullae were excluded. At the same time, a senior radiologist (who had 15 years of experience with thorax CT images) randomly selected 30 patients and repeated the delineation process. The Intraclass Correlation Coefficient (ICC) was used to evaluate consistency between observers.

### Feature extraction and selection

Image preprocessing and radiomics feature extraction were performed using python pyradiomics (version 3.0.1), which complies with IBSI (19). Image preprocessing includes resampling, denoising, and intensity standardization. Feature parameters include morphological features, first-order features, texture features, and transformation-based features. Before feature selection, radiomics features of different dimensions were normalized using a Z-score, which was used to remove the mean and variance normalization. The minimum Redundancy Maximum Relevance (mRMR) and the Least Absolute Shrinkage and Selection Operator (LASSO) were used for dimensionality reduction and feature selection.

### Model construction and evaluation

Univariate logistic regression analysis was used to screen independent clinical risk factors. According to the ratio of 7: 3, the patients were randomly divided into the training cohort and the validation cohort. The data of the training cohort were used to construct the model, and the data of the validation cohort were used to test. According to the radiomics labels and their weight coefficients, the radiomics score (Radscore) of every patient was calculated, and a radiomics model was established. Multivariate logistic regression analysis was used to establish a combined model based on radiomics features and clinical factors, and a nomogram was constructed. The area under curve (AUC) was calculated by receiver operating characteristic (ROC) curve analysis, and the performance of the training cohort and the validation cohort models was evaluated. The accuracy, sensitivity, specificity, Positive Predictive Value (PPV), and Negative Predictive Value (NPV) of the models were obtained. Delong test, Hosmer-Lemeshow test, and Decision Curve Analysis (DCA) were used to evaluate the diagnostic efficiency and clinical utility of the model.

### Statistical analysis

All data analysis was performed by using IBM SPSS version 24.0 (IBM Corp., Armonk, NY, USA). The continuous variables that conformed to be normally distributed were analyzed by the independent samples t-test. Otherwise, the continuous variables were analyzed by the Wilcoxon Rank-Sum test. The categorical variables were used the chi-square test or Fisher’s exact test. *P*< 0.05 was considered statistically significant.

## Results

### Patient characteristics

A total of 96 patients were included in this study. With the median follow-up of 59.1 months, 29 patients developed progression and 67 remained good controlled within two years. All patients were randomly assigned to the training cohort (n=68) and the validation cohort (n=28). There were no statistically significant differences in clinical factors between the training and validation cohorts (*P* > 0.05). Statistical characteristics were summarized in **Table 1**. Univariate logistic regression analysis showed that the type of peritumoral RILI was significantly different between the high-risk group and the low-risk group for disease progression (OR, 0.48; 95% CI: 0.25-0.90; *P*=0.022). Thus, a clinical model is established through this independent risk factor.

**Table 1:** Characteristics of patients in the training and validation cohorts.

	Training Cohort			Validation Cohort		
	High-risk (n=21)	Low-risk (n=47)	*p*	High-risk (n=8)	Low-risk (n=20)	*p*
Gender (%)						
Female	4 (19.0)	12 (25.5)		2 (25.0)	9 (45.0)	
Male	17 (81.0)	35 (74.5)	0.7849	6 (75.0)	11 (55.0)	0.58188
Age, years (mean ± SD)	74.5 (6.7)	73.6 (7.9)	0.6588	72.5 (6.6)	72 (9.2)	0.88141
KPS	88.8 (8.6)	89.8 (6.4)	0.6034	90 (7.6)	89.5 (7.6)	0.87475
Charlson	0.9 (1.2)	0.7 (1)	0.5613	1.6 (1.5)	0.6 (0.9)	0.02219*
Diameter, cm (mean ± SD)	2.3 (0.8)	2.5 (0.8)	0.2269	2.1 (0.7)	2.4 (0.9)	0.47013
Histology						
Adenocarcinoma	9 (42.9)	25 (53.2)		2 (25.0)	11 (55.0)	
Squamous cell carcinoma	10 (47.6)	13 (27.7)		3 (37.5)	4 (20.0)	
Not otherwise Specified	2 (9.5)	9 (19.1)	0.2404	3 (37.5)	5 (25.0)	0.34642
T stage						
1	16 (76.2)	34 (72.3)		4 (50.0)	17 (85.0)	
2	5 (23.8)	12 (25.5)		4 (50.0)	3 (15.0)	
3	0 (0.0)	1 (2.1)	0.7814	0 (0.0)	0 (0.0)	NA
Tumor location						
Central	2 (9.5)	3 (6.4)		1 (12.5)	0 (0.0)	
Peripheral	19 (90.5)	44 (93.6)	1.0000	7 (87.5)	20 (100.0)	0.62906
Involved lobe						
RLL/RML	8 (38.1)	15 (31.9)		2 (25.0)	8 (40.0)	
LLL	8 (38.1)	11 (23.4)		3 (37.5)	2 (10.0)	
LUL	2 (9.5)	7 (14.9)		2 (25.0)	4 (20.0)	
RUL	3 (14.3)	14 (29.8)	0.3922	1 (12.5)	6 (30.0)	0.31476
Pulmonary function						
Normal	3 (14.3)	4 (8.5)		1 (12.5)	1 (5.0)	
Mild	1 (4.8)	8 (17.0)		1 (12.5)	4 (20.0)	
Moderate	11 (52.4)	18 (38.3)		3 (37.5)	5 (25.0)	
Severe	6 (28.6)	17 (36.2)	0.3853	3 (37.5)	10 (50.0)	0.76868
Smoker						
No	6 (28.6)	21 (44.7)		4 (50.0)	10 (50.0)	
Yes	15 (71.4)	26 (55.3)	0.3241	4 (50.0)	10 (50.0)	1.00000
BED	98.1 (14.5)	98.5 (12.5)	0.9062	86.4 (14.8)	93.7 (19.3)	0.33499
BED≥100						
No	7 (33.3)	18 (38.3)		4 (50.0)	10 (50.0)	
Yes	14 (66.7)	29 (61.7)	0.9044	4 (50.0)	10 (50.0)	1.00000
Type						
1	10 (47.6)	34 (72.3)		5 (62.5)	14 (70.0)	
2	3 (14.3)	7 (14.9)		2 (25.0)	3 (15.0)	
3	8 (38.1)	6 (12.8)	0.0524	1 (12.5)	3 (15.0)	0.82186

KPS, karnofsky performance status; RLL, right lower lobe; RML, right middle lobe; LLL, left lower lobe; LUL, left upper lobe; RUL, right upper lobe; BED, biologically effective dose; Type, the type of peritumoral radiation-induced lung injury. *p< 0.05, expressive significance.

### Analysis based on CT radiomics features

#### Feature selection and model construction

**Figure 2:**
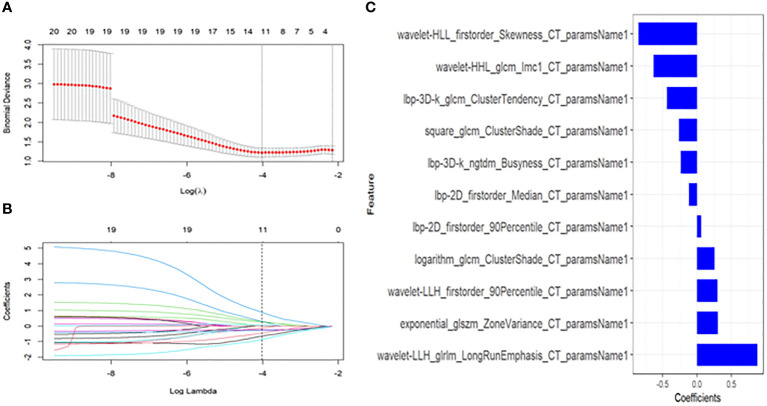
Textural feature selection using the Least Absolute Shrinkage and Selection Operator (LASSO) binary logistic regression. **(A)** Tuning parameters(λ) for the LASSO model were selected by 10-fold cross-validation using the minimum criteria. Partial likelihood deviance was plotted against log(λ). The dotted vertical lines correspond to the optimal values according to the minimum criteria and 1-SE criterion. The 11 features with the smallest binomial deviance were selected. **(B)** A feature coefficient convergence graph for filtering features using 10-fold cross-validation in the LASSO regression model. **(C)** LASSO coefficient profiles of texture features. Vertical lines correspond to the values selected by 10-fold cross-validation of the log(λ) sequence; the 11 nonzero coefficients are indicated.

**Figure 3:**
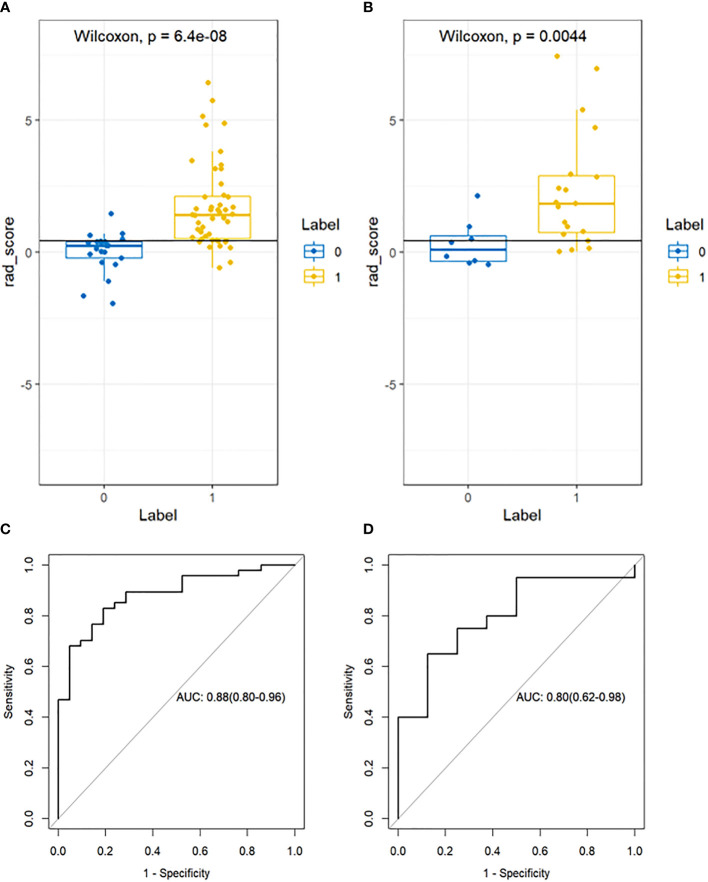
Box plot showing the Radscore distribution of high and low risk group for disease progression on training and validation cohorts. p-value from Wilcoxon Rank-Sum test **(A, B)**. Receiver Operator Characteristic (ROC) curves (training and validation cohorts) **(C, D)**. The prediction performance of the ROC curves for radiomics signature for training and validation cohorts.

A total of 1781 radiomics features (including morphological features, first-order features, texture features, and transformation-based features) were extracted from the pre-treatment CT images of early-stage NSCLC treated with SABR by using the python pyradiomics (version 3.0.1), ICC=0.82>0.75, indicating good inter-group consistency. After dimensionality reduction and feature selection by mRMR and LASSO, the 11 most valuable features and their corresponding coefficients were retained, as shown in **Figure 2**. The values of 11 features were input into the formula to obtain Radscore, and the radiomics model reflecting the disease progression was established. The box plot showed the Radscore distribution of high- and low-risk group for disease progression in training and validation cohorts, as shown in **Figure 3**. The resulting formula was as follows:

$$\begin{array}{l} \text{Radscore}=-0.12*\text{lbp}-2\text{D}\_\text{firstorder}\_\text{Median}+0.879*\text{wavelet}-\text{LLH}\_\text{glrlm}\_\text{LongRunEm}\\ \text{phasis}+-0.237*\text{lbp}-3\text{D}-\text{k\_ngtdm}\_\text{Busyness}+0.254*\text{logarithm}\_\text{glcm}\_\text{ClusterShade}+-0.\\ 266*\text{square\_glcm\_ClusterShade}+-0.852*\text{wavelet-HLL\_firstorder\_Skewness}+-0.635*\\ \text{wavelet-HHL\_glcm\_Imc}1+-0.442*\text{lbp-}3\text{D-k\_glcm\_ClusterTendency}+0.3*\text{exponential}\\ \text{l\_glszm\_ZoneVariance}+0.297*\text{wavelet-LLH\_firstorder\_}90\text{Percentile}+0.059*\text{lbp-}2\text{D\_}\\ \text{firstorder\_}90\text{Percentile}+1.212 \end{array}$$

**Figure 4:**
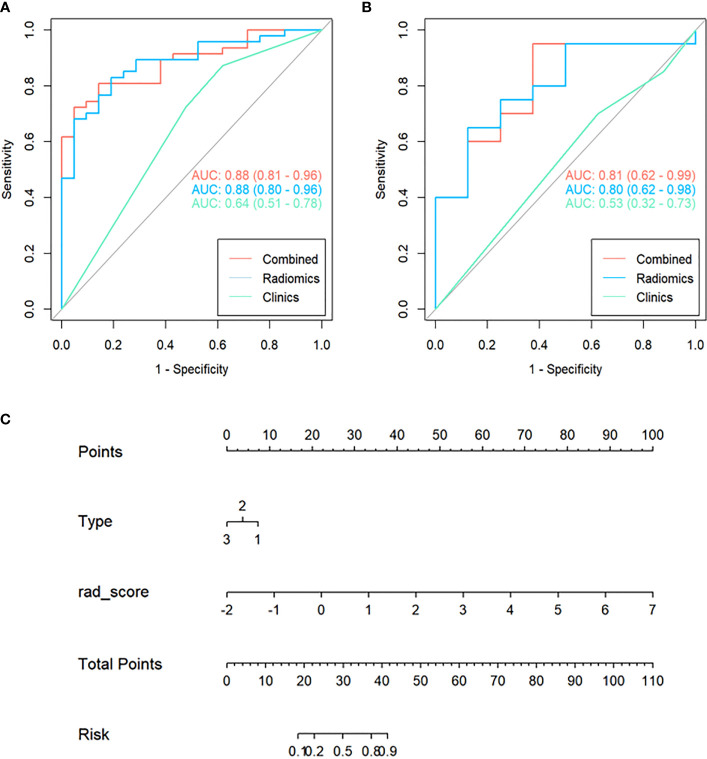
Receiver Operating Characteristic (ROC) curves of the clinical, radiomics, and combined model used to discriminate between the high and low risk of disease progression of lung cancer treated with SABR in the training and validation cohorts **(A, B)**. Radiomics nomogram **(C)** was used to discriminate the high and low risk of disease progression in lung cancer patients treated with SABR. The nomogram was based on the training cohort; the Radscore was shown. Initially, vertical lines were drawn at the Radscore values to determine the values of the points. The final point value was the sum of those of the two points. Finally, a vertical line was drawn at the total point value to determine the risk of disease progression of lung cancer treated with SABR.

Combined with the Radscore and the type of peritumoral RILI, a combined model was constructed, and a visual nomogram was formed, as shown in **Figure 4**.

#### Model performance evaluation

**Figure 4** showed that the AUC with its 95% confidence interval (CI) of the radiomics model, clinical model, and combined model was 0.88 (95%CI 0.80-0.96), 0.64 (95%CI 0.51-0.78), and 0.88 (95%CI 0.81-0.96) in the training cohort and 0.80 (95%CI 0.62-0.98),0.53 (95%CI 0.32-0.73) and 0.81 (95%CI 0.62-0.99) in the validation cohort, respectively. **Table 2** showed that the accuracy values of the radiomics model, clinical model, and combined model were 82.3%, 72.1%, and 79.4% in the training cohort, and 71.4%, 64.3%, and 85.6% in the validation cohort, respectively. The results showed that both the radiomics model and the combined model have good prediction efficiency in the training cohort and the validation cohort.

**Table 2:** Predictive performance of three prediction models for training and validation cohort.

Training cohort	AUC	95%CI	Sensitivity	Specificity	Accuracy	PPV	NPV
Clinical model	0.64	0.51-0.78	0.872	0.381	0.721	0.759	0.571
Radiomics model	0.88	0.80-0.96	0.830	0.810	0.824	0.907	0.680
Combined model	0.88	0.81-0.96	0.971	0.606	0.794	0.723	0.952
Validation cohort	AUC	95%CI	Sensitivity	Specificity	Accuracy	PPV	NPV
Clinical model	0.53	0.32-0.73	0.850	0.125	0.643	0.708	0.250
Radiomics model	0.80	0.62-0.98	0.750	0.625	0.714	0.833	0.500
Combined model	0.81	0.62-0.99	0.864	0.833	0.857	0.950	0.625

AUC, the area under the curve; CI, confidence interval; PPV, positive predictive value; NPV, negative predictive value.

**Figure 5:**
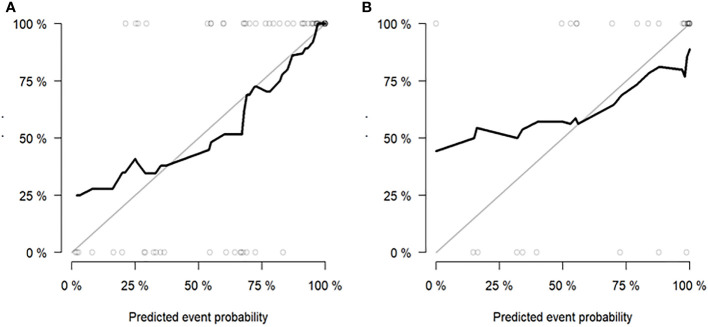
Hosmer-Lemeshow Test of the nomogram of the training **(A)** and validation **(B)** cohorts. The diagonal dotted lines represent the ideal predictions; the solid lines represent nomogram performance. A closer fit to the diagonal line indicates that the model matches better.

**Figure 6:**
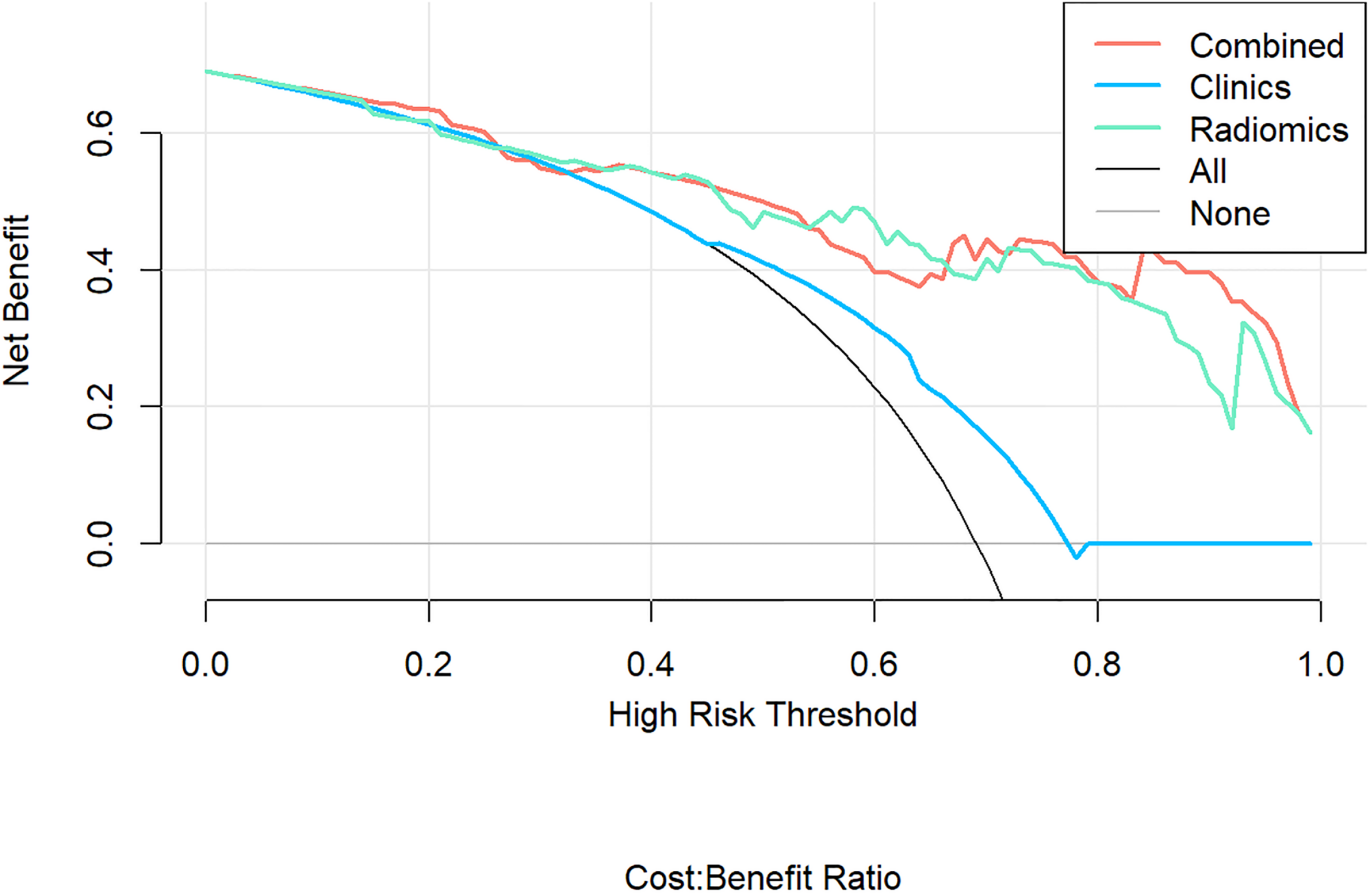
Decision Curve Analysis (DCA) results for the three discrimination models. The Y-axis represents the net benefit, calculated by summing the benefits (true positives) and subtracting the weighted harm (i.e., deleting false positives). The optimal method for feature selection is that with the highest net benefit.

According to the DeLong test, the performance of the radiomics model and combined model in the training and validation cohort was significantly better than that of the clinical model (*P*<0.05), but there was no statistically significant difference between the radiomics model and combined model (*P* > 0.05), as shown in **Table 3**. Hosmer-Lemeshow Test of the nomograms of the training and validation cohorts were shown in **Figure 5**, in which the results showed that the prediction of disease progression in the training cohort was well-calibrated (*P*>0.05). DCA results for the three discrimination models were shown in **Figure 6**. The results showed that the radiomics and combined models have high clinical benefits.

**Table 3:** Comparison of ROC curves with DeLong test in the training and validation cohort.

	Clinical vs Radiomics		Clinical vs Combined		Radiomics vs Combined	
	Z	*P*	Z	*P*	Z	*P*
Training Cohort	2.87	0.004*	3.48	<0.001*	0.093	0.926
Validation Cohort	2.08	0.038*	2.35	0.019*	0.24	0.812

*p< 0.05, expressive significance.

## Discussion

Radiomics can extract many disease features that cannot be observed with the naked eye from medical images and non-invasively capture information inside tumors that may be related to tumor recurrence, thereby realizing the goal of personalized medicine. In our study, a combined model based on pre-treatment thorax CT radiomics features and clinical factors was developed and validated to predict the likelihood of disease progression after SABR in early-stage NSCLC.

Distant metastasis was one of the main reasons for SABR treated in early-stage NSCLC. The RTOG 0236 study showed that the 5-year distant metastasis rate was 31% (18). In addition, the metastasis usually develops soon after the treatment of the primary lesion, and the survival time is significantly reduced once it occurs. For these patients with a high risk of early distant metastasis, systemic therapy combined with SABR may reduce the risk of metastasis and improve the OS. Therefore, it is of great significance to establish an accurate and effective predictive model to assess the risk of disease progression in patients of early-stage NSCLC.

Several studies have focused on the relationship between SABR prognosis and clinicopathological factors. Onishi et al. showed that BED ≥100Gy had significantly better local control rates and OS than those receiving BED< 100Gy (20). The predictive survival model showed that BED_10_< 113Gy was an independent risk factor for OS and PFS and was significantly associated with both local and distant progression (21). The prescription of BED ≥ 100Gy was currently recommended by international guidelines, including the National Comprehensive Cancer Network (NCCN) and the European Society of Medical Oncology (ESMO) guidelines. Kang et al. constructed a survival prediction model for stage I NSCLC treated with SABR, showing that tumor diameter >2.45 cm was an independent predictor of OS and PFS, which had a significant correlation with both local and distant progression (21). It is unclear whether there is any difference in the prognosis of different pathological types after SABR. Abel et al. analyzed 15,110 patients with early-stage (I ~ IIA) NSCLC who received SABR, and the 5-year OS of patients with adenocarcinoma and squamous cell carcinoma were 36% and 24% (*P*<0.0001), respectively. Squamous cell carcinoma was an independent poor prognostic factor (22). In our study, BED, tumor diameter, and pathological type did not correlate with disease progression, which may be related to the relatively concentrated BED dose (95% concentrated between 93.4Gy-99.4Gy), relatively uniform clinical factors, and a small number of cases and so on. Therefore, it is difficult to construct predictive models solely on the clinicopathological characteristics.

**Figure 7:**
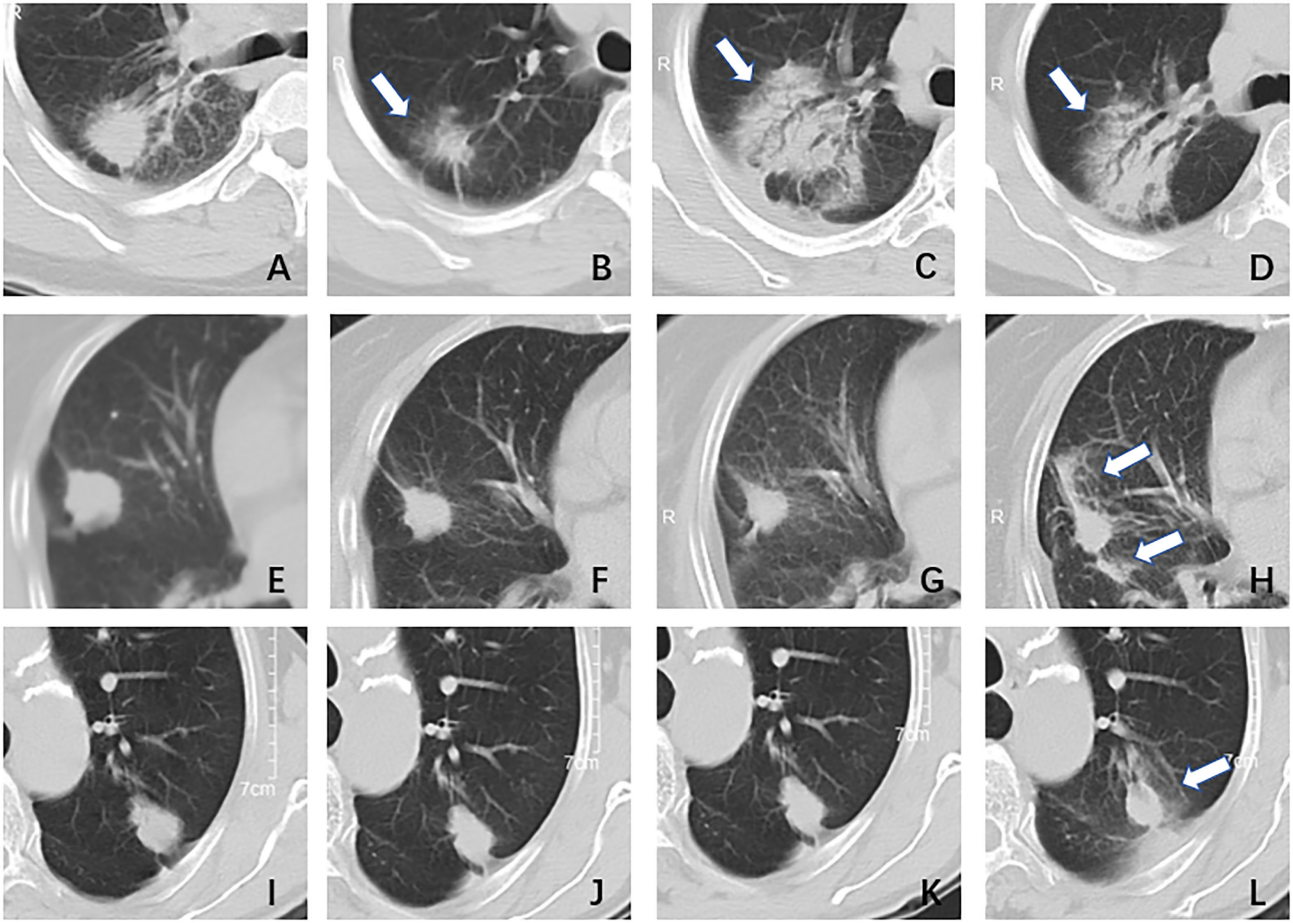
The type of peritumoral radiation-induced lung injury. Type I, female, 51 years, adenocarcinoma in the right lung, DT40GY/5F; **(A)** pre-treatment: a nodule with blurred boundary and spicule sign; **(B)** one month after treatment: the tumor shrunk and there was a surrounding ground-glass opacity; **(C)** three months after treatment: the tumor area showed diffuse consolidation and was indistinguishable from the tumor; **(D)** six months after treatment: the imaging findings were similar to **(C)**. Type II, female, 79 years, adenocarcinoma in right lung, DT55GY/5F; **(E)** pre-treatment: a nodule with a clear boundary and shallow lobed; **(F)**one month after treatment: the tumor has shrunk a little, no ground glass opacity surrounding it; **(G)** four months after treatment: there was no significant change; **(H)** six months after treatment: the tumor was surrounded by ground-glass opacity, more than 1/2. Type III, male,70 years, adenocarcinoma in left lung, DT50GY/5F; **(I)** pre-treatment: a nodule with a clear boundary and shallow lobed; **(J)** two months after treatment: there was no significant change; **(K)** four months after treatment: there was no significant change; **(L)** six months after treatment: the tumor was surrounded by ground-glass opacity, less than 1/2.

The type of peritumoral RILI was the only independent risk factor for tumor progression among clinical factors (*P*< 0.05). The pattern of changes in lung parenchyma on CT post-SABR can generally be categorized as acute (within six months, corresponding to pneumonitis) or late (after six months, corresponding to fibrosis) (23). Several papers have classified acute changes into one of five general patterns: diffuse consolidation, patchy consolidation, diffuse ground-glass opacities (GGO), patchy GGO, and no change (24–26). In the past, the vast majority of literature discussed the identification of RILI and tumor recurrence (27–29), and there were few studies on the correlation between them. Based on the above considerations, we redefined the peritumoral RILI and divided them into three types (**Figure 7**). Type I is diffuse consolidation around the tumor, also called severe RILI. Type II is diffuse GGO around the tumor, which is distributed over 180 degrees around the tumor; we also call it moderate RILI. Type III is patchy GGO within a range of fewer than 180 degrees around the tumor, or there is no change; we call it mild RILI.

In this study, a total of 1781 radiomics features were extracted. After screening of radiomics features by mRMR and LASSO, 11 features were finally retained, including four first-order features and seven texture features, all based on transformation. First-order features describe the gray value distribution of tumor image ROIs. In this study, Skewness reflects the asymmetry of gray value distribution relative to the mean. The more low-signal gray distribution in the lesion is, the higher the tumor heterogeneity.

In the texture feature, the Gray Level Co-occurrence Matrix (GLCM) studies the spatial correlation characteristics between the gray levels of two points in a certain distance and direction in the image so as to reflect the texture information of the image in direction, interval, change amplitude and speed. In this study, Informational Measure of Correlation (IMC) 1 assesses the correlation between the probability distributions of i and j (quantifying the complexity of the texture); Cluster Tendency is a measure of groupings of voxels with similar gray-level values; Cluster Shade is a measure of the skewness and uniformity of the GLCM, a higher cluster shade implies more significant asymmetry about the mean. The Gray Level Run Length Matrix (GLRLM) mainly reflects texture roughness and directionality. It is used to describe the length of the same pixel gray level that appears continuously in a specified direction. In this study, Long Run Emphasis (LRE) measures the distribution of long-run lengths, with a more excellent value indicative of long run lengths and more coarse structural textures. The Gray Level Size Zone Matrix (GLSZM) provides information about the spatial distribution of corresponding adjacent pixels or voxels at the same gray level. In this study, Zone Variance (ZV) measures the variance in zone size volumes for the zones, and the more significant the value, the greater the heterogeneity. The Neighbouring Gray Tone Difference Matrix (NGTDM) represents the difference between the gray value of a point and the average gray value in the neighborhood at a certain distance, thereby capturing the spatial rate of gray intensity changes. In this study, busyness is a measure of the shift from a pixel to its neighbor; a high value for busyness indicates a ‘busy’ image, with rapid changes of intensity between pixels and their neighborhood. The gray information of these images can quantitatively analyze tumor heterogeneity so as to conduct quantitative studies at the microscopic level, which can effectively predict the disease progression of patients (30, 31). The features selected in this study were all processed by filters, which may be because filters can extract and reconstruct the parts of the original images, thus mining deeper image information.

In this study, the AUC of the radiomics model and combined model in the training and validation cohorts were all above 0.80, and the accuracy rates were above 0.7. The model we developed showed a good predictive efficiency of disease progression after SABR, which provided important information for subsequent clinical therapy and follow-up. Rainer et al. also had similar findings. This study predicted tumor progression six months after Stereotactic Body Radiation Therapy (SBRT) for early-stage lung cancer, enrolled 399 patients from 13 different units, and finally retained seven radiomics features to establish a Support Vector Machine (SVM) model, using 10-fold cross-validation and AUC to evaluate the performance of the classifier. The results showed that the AUC was 0.789, sensitivity was 67.0%, and specificity was 78.7%, which was a good prediction (32). Lafata et al. also proposed the potential relationship between radiomics features extracted from pre-treatment CT images and clinical outcomes following SBRT for NSCLC; the results showed that two features demonstrated a statistically significant association with local failure: Homogeneity2 (*p*=0.022) and Long-Run-High-Gray-Level-Emphasis (*p*=0.048) multivariable logistic regression models produced AUC values of 0.83 (33).

In the DeLong test, we found no statistically significant difference between the radiomics model based solely on the CT images and the combined model, which indirectly confirmed the dominant role of CT images in the prediction model. Even so, compared with the ROC, the AUC value of the combined model is higher than that of the pure CT radiomics model. Therefore, clinical variables (the type of peritumoral RILI) still have a specific positive effect on the comprehensive judgment of the model. Luo et al. and Li et al. also proposed that clinical variables were significantly correlated with the clinical outcomes of patients receiving SBRT for lung cancer and proved that the combined model based on clinical factors and radiomics features could effectively improve model prediction efficiency (34, 35).

Limitations of this study: Firstly, this study was a retrospective study, which can only be analyzed based on existing data, and prospective studies can be carried out in the future to incorporate some new variables. Secondly, the number of cases in this study was limited, and the sample size needed to be further expanded to improve the stability of the model. Thirdly, the data in this study came from the same hospital, and only internal validation was performed. Data from other hospitals should be added for external validation to improve model repeatability.

## Conclusions

In conclusion, the radiomics model established based on pre-treatment thorax CT images of early-stage NSCLC can predict the disease progression after SABR treatment. At the same time, the nomogram we developed has a better predictive ability for the disease progression and provides a feasible and practical reference value for clinical guidance of individualized treatment, follow-up, and evaluation strategies for patients undergoing SABR.

## Data availability statement

The raw data supporting the conclusions of this article will be made available by the authors, without undue reservation.

## Ethics statement

The studies involving human participants were reviewed and approved by Ethics committees of Cancer Hospital of the University of Chinese Academy of Sciences. Written informed consent for participation was not required for this study in accordance with the national legislation and the institutional requirements.

## Author contributions

HY had full access to all of the data in the study and took responsibility for the integrity of the data and the accuracy of the data analysis. HY and LW are co-first authors of this article. Collection and assembly of data: BD, XD, and HM. Resources data curation: XS, ZW, XZ, XL, WF, LS, GQ, JL, JZ, CW, QZ, XY, QC, MC, PL, YW, YJ, JC,YJ and XH. Data analysis and interpretation: GS, FW, HC, WC, YZ,YH and YZ. Writing-original draft preparation: HY and LW. Statistical analysis: HY, YGW. Study concept, supervision, funding acquisition, project administration, and writing-review: HJ and YX. All authors contributed to the article and approved the submitted version.

## Funding

This study was supported by grants from Medical and Health Research Project of Zhejiang Province (Grant Number: 2020KY486 ; 2020KY079); Beijing Xisike Clinical Oncology Research Foundation (Y-2019AZMS-0061); and Beijing Science and Technology Innovation Medical Development Foundation (KC2021-JX-0186-63).

## Conflict of interest

The authors declare that the research was conducted in the absence of any commercial or financial relationships that could be construed as a potential conflict of interest.

## Publisher’s note

All claims expressed in this article are solely those of the authors and do not necessarily represent those of their affiliated organizations, or those of the publisher, the editors and the reviewers. Any product that may be evaluated in this article, or claim that may be made by its manufacturer, is not guaranteed or endorsed by the publisher.
